# Development of Hybrid Materials Based on Chitosan, Poly(Ethylene Glycol) and Laponite^®^ RD: Effect of Clay Concentration

**DOI:** 10.3390/polym15040841

**Published:** 2023-02-08

**Authors:** Simona Morariu, Cristina-Eliza Brunchi, Mirela Honciuc, Manuela-Maria Iftime

**Affiliations:** “Petru Poni” Institute of Macromolecular Chemistry, 700487 Iasi, Romania

**Keywords:** Laponite^®^ RD, poly(ethylene glycol), chitosan, hybrid materials, aqueous dispersions, rheological properties, nanocomposite films

## Abstract

In the context of increasing interest in biomaterials with applicability in cosmetics and medicine, this research aims to obtain and characterize some hybrid materials based on chitosan (CS) (antibacterial, biocompatible, and biodegradable), poly(ethylene glycol) (PEG) (non-toxic and prevents the adsorption of protein and cell) and Laponite^®^ RD (Lap) (bioactive). The rheological properties of the starting dispersions were investigated and discussed related to the interactions developed between components. All samples exhibited gel-like properties, and the storage modulus of CS/PEG dispersion increased from 6.6 Pa to 657.7 Pa by adding 2.5% Lap. Structural and morphological characterization of the films, prepared by solution casting method, was performed by Fourier transform infrared spectroscopy (FTIR), scanning electron microscopy (SEM), energy dispersive X-ray spectroscopy (EDX), and polarized light microscopy (POM). These analyses proved the incorporation of Lap into CS/PEG films and revealed the morphological changes of the films by the addition of clay. Thereby, at the highest Lap concentration (43.8%), the “house of cards” structure formed by Lap platelets, which incorporate chitosan chains, as evidenced by SEM and POM. Two stages of degradation between 200 °C and 410 °C were evidenced for the films with Lap concentration higher than 38.5%, explained by the existence of a clay-rich phase (given by the clay network) and chitosan-rich one (due to the intercalation of chitosan in the clay network). CS/PEG film with 43.8% Lap showed the highest swelling degree of 240.7%. The analysis of the obtained results led to the conclusion that the addition of clay to the CS/PEG films increases their stability in water and gives them greater thermal stability.

## 1. Introduction

The hydrogel or film nanocomposites based on polymer/clay mixtures have a great potential for a variety of applications including drug delivery [1], wound dressing [2], bone tissue engineering [3], active food packaging [4], contaminant adsorption [5,6]. For such applications, it is preferable to use natural polymers which have low cost due to their abundance in nature, low toxicity, and biodegradable and biocompatible nature. Chitosan (CS) is a cationic polysaccharide from the natural environment frequently used in the designing of bio-nanocomposite materials. The traditional method to obtain CS is based on the processing of waste crustaceans’ shells but, in the last years, many efforts were directed at its obtaining from the fungal kingdom [7]. The structure of CS is composed of β–(1–4)–linked D–glucosamine and N–acetyl–D–glucosamine units, randomly distributed along the polymer chain, and presents –NH_2_ groups in its structure that give it cationic nature. In addition, CS possesses in its structure –OH groups able to attract the positively charged molecules [8]. These functional groups allow the modification of the CS structure improving its properties. The main applications of CS are in the medical field due to its biomedical properties: antimicrobial, antioxidant, and anti-inflammatory activity, and hemocompatibility. Thereby, it is used in the design of membranes, hydrogels, sponges and fiber materials in skin tissue engineering [9]. Although CS has been found to have remarkable biological properties required for medical applications, its weak mechanical properties limit the range of applications. The mixing of CS with clay leads to materials with superior mechanical properties, thereby widening the range of applications. The hybrid materials that combine the properties of the pure components are obtained by mixing cationic CS with smectite clays containing anionic centers. In an acidic environment (pH < 6.5), the amino groups on CS chains are protonated favoring their electrostatic interactions with negatively charged clay surfaces covering the clay particles. In addition, CS chains can penetrate between the clay interlayers via a cationic exchange process [10].

CS/clay nanocomposites, where CS is in excess, can be used in the potentiometric detection of anions species [11]. Montmorillonite (MMT) is the most used clay from the smectite class to prepare CS/clay hydrogels or films. Recently, Altunkaynak et al. [12] have prepared, by solvent casting method, CS/MMT composite films which can be used as controlled release systems of Paroxetine Hydrochloride. The composite films containing the sodium form of MMT exhibited a higher percentage of released drugs due to the increase of space between silicate layers, determining the increase of swelling capacity. Nanocomposites based on CS and clay were investigated as drug delivery carrier for release of various drugs: ibuprofen [13], chlorphenamine [14], pantoprazole [15], diclofenac sodium [16], glucantime [17], etc. The thermal stability of CS/MMT nanocomposites is improved by increasing the clay amount [18]. Recently, it was found that nanocomposites based on CS and MMT, loaded with vanillin and cinnamaldehyde as natural antioxidants, favor the growth of tomato seedlings [19]. The addition of MMT into carboxymethyl chitosan hydrogel confers them the good flame retardant properties and excellent sound-adsorption capacity due to their low density (33.85–38.27 mg/cm^3^) and high porosity (98.20%) [20]. The biodegradability and antimicrobial activity of the CS/clay nanocomposites can be enhanced by adding a synthetic biopolymer, such as, poly(vinyl alcohol) (PVA), poly(ethylene oxide) (PEO), poly(acrylic acid), poly(lactic acid), poly(vinylpyrrolidone), etc. [21]. pH responsible biocomposites based on CS and PVA were designed by using a layered double hydroxides (LDH) anionic clay [22]. These biocomposites showed the best values of viscoelastic parameters at pH close to the neutral one and 37 °C (physiological conditions) [23].

Films for the food packaging, with color change properties as a function of pH, were tailored by using CS and biohybrid complexes consisting of anthocyanins adsorbed on Laponite^®^ surface [24]. The tests for monitoring the freshness of the meat at various temperatures showed the alteration of the meat by changing the color from purple to yellow. Laponite^®^ RD (Lap) is a hectorite clay with a 2:1 lamellar structure (an octahedral sheet between two tetrahedral sheets) belonging to the smectite group. Similar to any smectite clay, Lap has the ability to swell in water, where clay nanoparticles are platelets with a diameter of ca. 25 nm and a thickness of 1 nm [25]. In aqueous dispersion, Lap disks have a strongly negative charge on the faces and a weakly positive charge on the edges [26]. Lap can act as a physical crosslinker due to its capacity to interact with polymer chains by a mechanism that is still discussed. PEO/Lap/water systems are the most widely investigated in order to establish the polymer adsorption mechanism onto Lap platelets [27,28,29,30,31,32,33,34,35,36].

It was proven that the addition of Lap into natural or synthetic polymer material gives it bioactive and osteoinductive properties, in addition to superior mechanical properties, due to its ability to interact with various molecule types or enzymes [37,38,39]. Thereby, the incorporation of a small amount of Lap into the PVA hydrogel improved antibacterial activity against Gram-positive and -negative bacteria [40] and the Lap/PVA hydrogels could be used in designing of wound dressings [41]. Schmidt and coworkers [42,43] have developed CS/PEO/Lap gels for medical use, combining the capacity of PEO to prevent protein and cell adhesion with that of promoting cell growth and adhesion of CS and the bioactive property of Lap. The increase of CS amount in gels determines the increase of rheological parameters but diminishing of the elastic recovery capacity [44].

Previously, we have shown that the addition of up to 0.453% CS into Lap (2.8%)/PEO (2.8%) hydrogels determines the improvement of their rheological properties due to the intensification of the interactions between the three components [44]. Despite the large literature, due to the multiple possibilities of interaction of Lap with polymers (depending on the structure and concentration of the components or the experimental conditions), many aspects of this topic are still debated. In the present paper, this approach is extended to explore the effect of Laponite^®^ RD addition on the structure, morphology, and properties of chitosan/poly(ethylene glycol) films prepared by the solution casting method.

## 2. Materials and Methods

### 2.1. Materials

Chitosan (CS) (degree of acetylation of 26%) and poly(ethylene glycol) (PEG) (polydispersity index of l.04) samples with molecular weights of 7.14 × 10^5^ g/mol [45] and 400 g/mol, respectively, were purchased from Sigma-Aldrich Chemie GmbH (Taufkirchen, Germany). Laponite^®^ RD (Lap), with chemical formula Na^+^_0.7_[(Si_8_Mg_5.5_Li_0.3_)O_20_(OH)_4_]^−0.7^, has been donated by Rockwood Additives Limited U.K. (now BYK Additives Ltd., Widnes, UK). Acetic acid (99.8% purity) and NaOH were also purchased from Sigma-Aldrich Chemie GmbH, Germany. All materials were used without further purification. Millipore water, obtained from a Milli-Q PF apparatus (Millipore, Switzerland), was used for the samples preparation and their swelling.

### 2.2. Dispersions and Films Preparation

Different amounts of Laponite^®^ RD were dispersed in Millipore water by magnetic stirring for 15 min. Then, about 0.6 g PEG was added by vigorous stirring for 30 min, followed by ultrasonication for 30 min in order to obtain stable dispersions. Finally, certain amounts of 1% CS solution in 1% acetic acid solution and of 0.1 M NaOH solution were added alternatively drop by drop to reach the desired chitosan concentration and a pH of 7.3 (physiological condition). CS solutions were obtained after vigorous stirring at room temperature for 2 h. These dispersions were used in turbidity and rheological measurements. The dispersions were poured into plastic Petri dishes and dried at room temperature for 5 days to form films. FTIR, SEM, POM, thermal analysis, and swelling measurements were taken on the films prepared by the solution casting method. The compositions of dispersions and final films are shown in Table 1. The concentrations are expressed in wt/wt %.

### 2.3. Rheological Investigations

The rheological tests were performed with a Bohlin CVO Rheometer, at 37 °C, by using parallel plate geometry with a diameter of 60 mm. The temperature control is ensured by the Peltier effect. The amplitude sweep measurements were carried out at an oscillation frequency (ω) of 1 rad/s by varying the shear stress, τ, from 0.01 Pa to 100 Pa, in order to determine the linear viscoelastic regime (LVR) where storage (G′) and loss (G″) moduli do not depend on τ. The oscillatory tests were performed in the range of ω *between* 0.03 rad/s and 200 rad/s at a τ value from LVR. The apparent viscosity (η_app_) was established for all samples from the measurements in the continuous shear regime, carried out at the shear rates ($\dot{\gamma }$) up to 500 l/s.

### 2.4. Turbidimetry Measurements

The turbidity measurements were made on a HACH 2100AN turbidimeter in the range of 400–600 nm wavelengths at room temperature. The turbidimeter was calibrated with StablCal^®^ Stabilized Formazin Standards, 0–7500 NTU, before the measurements. HACH 2100AN turbidimeter is equipped with a 90-degree detector, a forward scatter light detector, a back scatter detector and a transmitted light detector. Ratio mode allows the turbidity determination up to 10,000 NTU with the following relationship:

(1) $$Turbidity(NTU)=\frac{I_{90}}{a_{1}\cdot I_{fs}+a_{2}\cdot I_{bs}+a_{3}\cdot I_{t}+a_{4}\cdot I_{90}}$$

where: a_1_, a_2_, a_3_ and a_4_ are calibration coefficients, I_90_, I_fs_, I_bs_ and I_t_ represent 90-degree detector current, forward scatter detector current, back scatter detector current, and transmitted detector current, respectively.

Signal Averaging mode gives one value which is an average of 10 measurements. The accuracy of the data given by the turbidimeter in the Nephelometric Turbidity Unit (NTU) was ±2% for 0–1000 NTU and ±5% for 1000–4000 NTU.

### 2.5. Fourier Transform Infrared Spectroscopy (FTIR)

FTIR spectra of the pure components and films were obtained at room temperature with an IRAffinity-1S Fourier Transform Infrared Spectrometer (Shimadzu UK Ltd., Milton Keynes, UK). The spectra were recorded in the range of 4000–500 cm^−1^ with a resolution of 2 cm^−1^.

### 2.6. Scanning Electron Microscopy (SEM)

The morphology of the film’s surface was investigated with Verios G4 UC Scanning Electron Microscope (SEM) (FEI Company, Hillsboro, OR, USA) type Quanta 200, coupled with an energy dispersive spectrometer (EDS, EDAX Octane Elite) for determination of elemental composition. SEM micrographs with high magnification were obtained at an accelerated electron energy of 5 kV.

### 2.7. Polarized Light Microscopy (POM)

The organization of the polymer chains and clay platelets into films was investigated by polarized light microscopy (POM) on the samples placed between two lamellae, by using a Zeiss Axio Imager.A2m optical microscope. All images were registered using an Axiocam 208cc camera. (Carl Zeiss AG, Oberkochen, Germany). For SEM and POM, more images were captured from different areas of the samples to obtain the most suggestive images of the film microstructure.

### 2.8. Thermogravimetric Analysis (TGA)

Thermogravimetric analysis (TGA) and derivative thermogravimetry (DTG) measurements were performed with a TA Instruments TGA 5500 thermogravimetric analyzer. The thermal stability of the films was explored over a temperature range from 30 °C to 700 °C at a heating rate of 10 °C/min.

### 2.9. Swelling Experiments

The swelling degree, S, of CS/PEO/Lap films, was determined in Millipore water at room temperature. After removing from the water, the samples were gently wiped out with an absorbent paper and weighed. The swelling degree was determined by the following relationship:

(2) $$S=\left(w_{t}-w_{0}\right)/w_{0}\cdot 100\%$$

where w_0_ is the weight of the dried hydrogel and w_t_ represents the hydrogel weight at the immersion time t.

### 2.10. Statistical Analysis

The experimental data fitting and the statistical analysis were performed using OriginPro8.5 software (OriginLap Corporation, Northampton, MA, USA). Zero shear viscosity values are presented as mean ± SE (standard error of the mean values) estimated from the nonlinear regression of the three parameter Carreau model. The values of the swelling degree are also given as mean ± SE based on three independent experiments. The differences between the means of swelling degree, performed with One-way ANOVA (Tukey HSD analysis), were not significant at *p* ≤ 0.05.

## 3. Results and Discussions

**Scheme 1:**
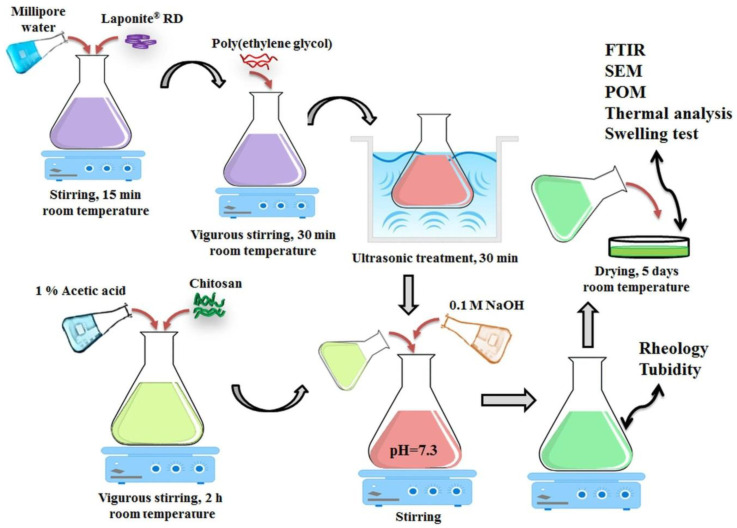
Illustration of the experimental steps of film preparation.

The investigated films were prepared according to Scheme 1.

### 3.1. Rheological Properties of CS/PEG Dispersions with/without Lap

Knowing the rheological properties of the starting dispersions plays an important role in understanding the interactions developed between the components in aqueous medium, and as well as the subsequent processing of the films.

**Figure 1:**
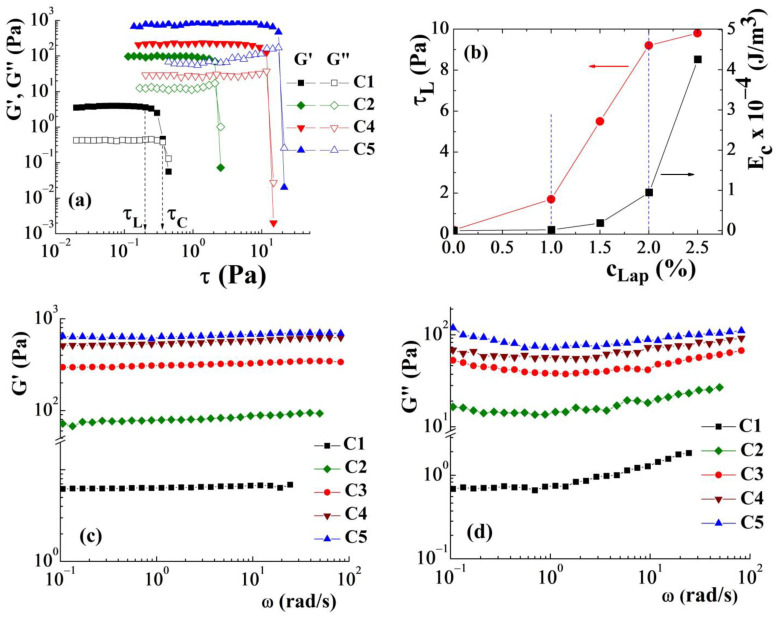

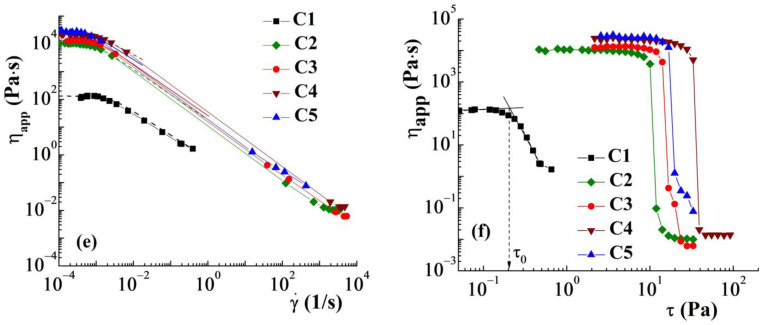
Effect of Lap addition on the rheological behavior of CS/PEG/Lap aqueous dispersions: (**a**) the viscoelastic moduli, G′ and G″, *vs*. shear stress, τ, at 1 rad/s and 37 °C; (**b**) variation of τ_L_ and E_c_ as a function of clay concentration, c_Lap_; (**c**) the storage modulus, G′ and (**d**) the loss modulus, G″ *vs*. oscillation frequency, ω at 37 °C; the apparent viscosity, η_app_, *vs*. (**e**) shear rate, $\dot{\gamma }$ (the full lines represent the fits of experimental data with the three-parameter Carreau equation) and (**f**) shear stress, τ.

The amplitude sweep tests allowed the establishment of the linear viscoelastic regime (LVR) for each studied sample, where storage (G′) and loss (G″) moduli are independent of the applied stress (Figure 1a). The investigated samples exhibit gel-like properties with storage modulus, G′, greater than loss modulus, G″, due to the formation of physical networks between components. G′ and G″ remain constant up to a limit value of shear stress (τ_L_), from which the network structure starts to break and, after a critical value, τ_C_, G″ becomes higher than G′ (liquid-like behavior). As can be seen in Figure 1a,b, the addition of Lap expands the linear viscoelastic domain. Thereby, LVR increases by adding Lap from 0.2 Pa for C1 to about 10 Pa for C5. The viscoelastic moduli, G′ and G″, also increase by approximately two orders of magnitude by adding 2.5% clay (Table 2).

Considering the strain which limits LVR (denoted with γ_L_) and G′ value corresponding to γ_L_ (denoted with $G_{o}^{\prime }$), the cohesive energy density (E_c_) can be estimated with the following equation [47]:

(3) $$E_{c}=\frac{1}{2}G_{o}^{\prime }\gamma_{L}^{2}$$

The value of E_c_ can be related to the interactions between particles and/or polymer chains in aqueous dispersions [48]. The higher values of E_c_ are given by the stronger interactions in dispersion. Three domains can be delimited in the variations of τ_L_ and E_c_ as a function of Lap concentration, c_Lap_ (Figure 1b): (i) below 1% Lap when E_c_ increases from about 0.1 J/m^3^ for CS/PEG dispersion free of Lap to 172.2 J/m^3^ for CS/PEG/Lap dispersion with 1% Lap; (ii) 1% < c_Lap_ < 2%, when E_c_ increases with an order of magnitude by increasing c_Lap_ from 1% to 2% and LVR increases significantly; (iii) above 2% Lap, when E_c_ increases from 9479.7 J/m^3^ for 2% Lap to 42,574.5 J/m^3^ for 2.5% Lap while the LVR does not change significantly. The rheological observations can be correlated with the phase diagram of Lap in water. Thereby, neat clay aqueous dispersions (without other additions) exhibit the following phases: (i) below 1% Lap, phase separation occurs; (ii) for 1% < c_Lap_ < 2%, an attractive gel composed mainly of individual Lap platelets is formed; (iii) 2% < c_Lap_ < 2.8%, the attractive gel is composed more of small tactoids of Lap disks; (iv) for c_Lap_ above 2.8%, a nematic gel is distinguished [49,50]. The structure of Lap aqueous dispersion is continuously rearranged to reach the minimum energy state. The addition of PEG induces changes in the Lap platelets arrangement and, a slowing down of the aggregation process occurs due to the adsorption of short chains of polymer on the clay particles’ surface, preventing the establishment of edge–face interactions [51].

The frequency sweep tests were performed at 37 °C at shear stress values from LVR corresponding to each sample (0.1 Pa for C1, 1 Pa for C2, 5 Pa for C3, C4 and C5). In Figure 1c,d is shown the effect of c_Lap_ on the viscoelastic moduli of CS/PEG/Lap aqueous dispersions. G′ values remain constant on the whole investigated frequency range, irrespective of the added Lap amount, due to the strong networks which are not affected by the shear. G″ is affected differently by shear, depending on c_Lap_ in the sample. Thereby, in the absence or low concentration of clay, G″ value increases slightly at ω values higher than about 1 rad/s due to structural disorder and metastability of the samples [52]. For the dispersions with higher amounts of Lap (C3, C4 and C5), G″ also increases slightly at small ω values, showing a minimum around 1 rad/s. This behavior could be related to aging phenomena characteristic for the Lap dispersions [53]. All dispersions exhibit gel-like properties with G′ greater than G″ and values of tan δ, defined as G″/G′, lower than unity (Table 2).

The continuous shear measurements revealed the decrease of apparent viscosity, η_app_, by increasing the shear rate (shear–thinning behavior), $\dot{\gamma }$, behavior characteristic of the pseudoplastic materials. The flow curves of samples and the value of zero shear viscosity (η_0_) determined by using the three parameter Carreau equation, are shown in Figure 1e and Table 2, respectively. One can observe that the addition of Lap determines the increase of η_0_ from 131.7 Pa·s for CS/PEG dispersion without Lap to 28,875 Pa·s for that with 2.5% Lap. Yield stress values, τ_0_, were determined as being the shear stress at which the viscosity suddenly decreases, and the sample starts to flow (Figure 1f). A slight increase of τ_0_ was evidenced by Lap addition (Table 2) as a result of the intensification of the interactions between the dispersion components.

### 3.2. Turbidimetric Analysis

**Figure 2:**
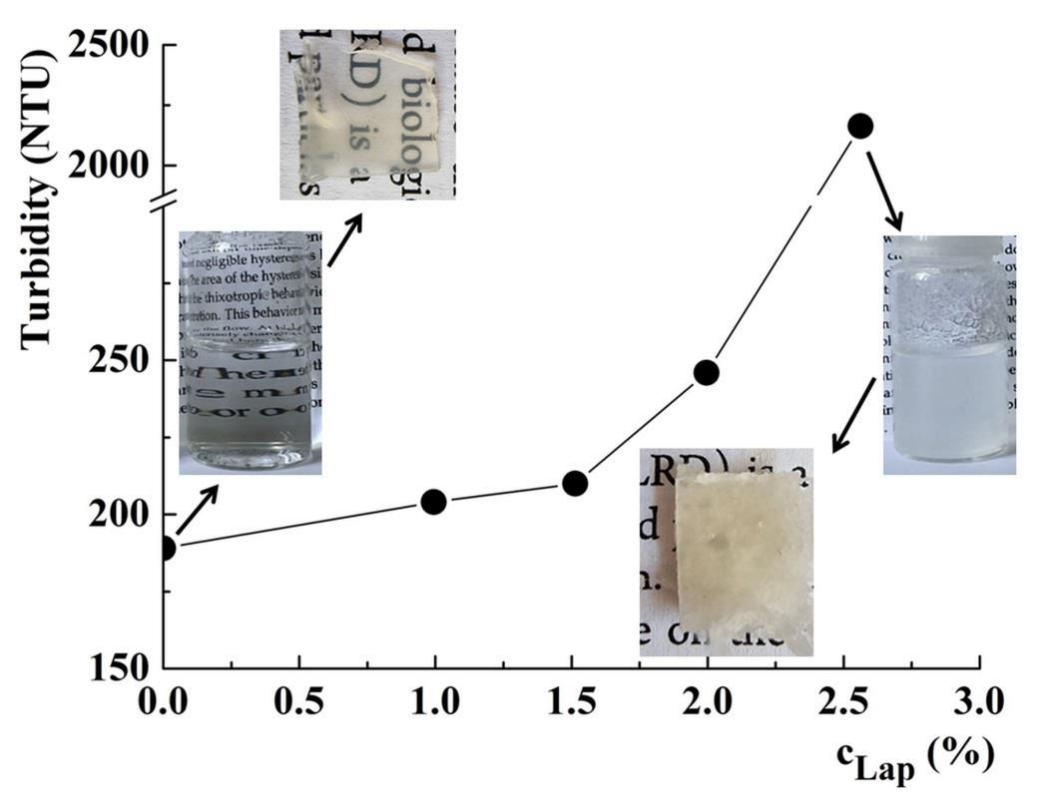
Effect of c_Lap_ on the turbidity of dispersions with 3% PEG and 0.2% CS; visual appearance of dispersions/films free of Lap and with 2.5% Lap.

The turbidimetric measurements show a slight increase from 189 NTU for the dispersion free of Lap to 286 NTU for the dispersion with 2% Lap (Figure 2).

The further increase of c_Lap_ to 2.5% causes a sudden increase in turbidity to 2163 NTU. The film’s transparence decreases as the clay content increases. The increase of opacity of dispersion/film could be due to the strong interactions between the components and the formation of CS–Lap and PEG–Lap complexes.

### 3.3. Structural and Morphological Characterization of Films

**Figure 3:**
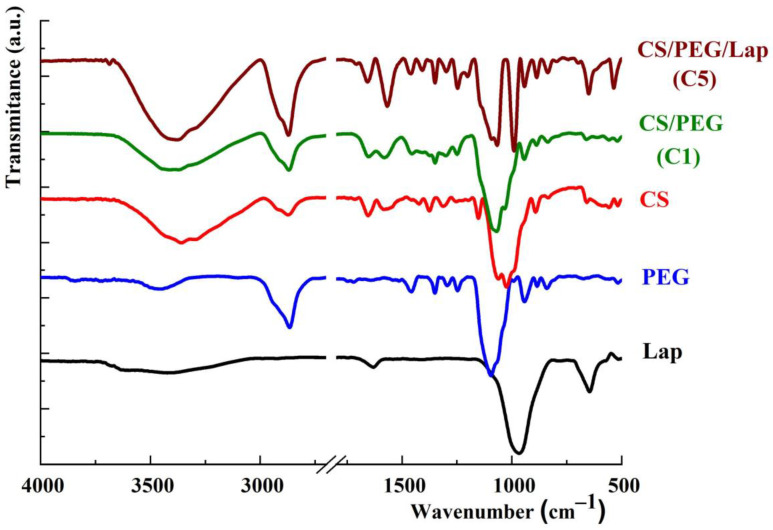
FTIR spectra of CS/PEG films with/without Lap and the pure components.

In Figure 3 are illustrated the FTIR spectra of CS/PEG films with and free of clay compared to those of their components. In Lap spectrum are identified two intense bands related to Si–O and Mg–O groups at 966 cm^−1^ and 646 cm^−1^, respectively. The weak bands at 3614 cm^−1^, 3416 cm^−1^ and 1630 cm^−1^ correspond to the –OH stretching from Si–OH group, –OH stretching from free water and, water bending modes [44,54]. For pure PEG, the main peaks are observed at wavenumbers of 3450 cm^−1^ (OH stretching), 2868 cm^−1^ (CH stretching), 1458 cm^−1^ (CH bending vibrations from CH_2_ groups), 1248 cm^−1^ (C–O stretching vibration). The peak due to C–O–C symmetrical stretching is observed at 1095 cm^−1^ [55,56]. In FTIR spectrum of pure CS, a broad peak around 3350 cm^−1^ (3640–2960 cm^−1^) is due to the overlapping of OH and NH stretching vibrations and the hydrogen bonding between the CS chains. In addition, absorption bands are observed at 2874 cm^−1^ and 1425 cm^−1^ which correspond to the vibration of pyranose ring (CH_2_ stretching and OH/CH vibrations). The peaks identified at 1654 cm^−1^ and 1560 cm^−1^ are due to stretching vibration of the C=O bending from –NHCO– groups (amide I) and N–H vibration of –NH_2_ groups (amide II). The bands for stretching vibration of C–O–C group in glycosidic linkage are observed between 910 cm^−1^ and 1130 cm^−1^ (1153 cm^−1^, 1060 cm^−1^ and 1026 cm^−1^) [57,58].

In CS/PEG spectrum, the band corresponding to OH and NH stretching vibrations is shifted from 3350 cm^−1^ in CS spectrum to about 3400 cm^−1^. Moreover, it was observed the shift of the peak corresponding to C–O–C group of chitosan from 910–1130 cm^−1^ to 980–1180 cm^−1^. These changes suggest the formation of new intermolecular interactions between CS and PEG by hydrogen bonds. The bands corresponding to amide I and amide II groups from CS are not shifted but the appearance of the peaks is changed and peaks increase in intensity. In addition, the peak attributed to C=O bending from –NHCO– groups is slightly shifted from 1654 cm^−1^ to 1649 cm^−1^ due to the additional interactions between CS segments and OH groups of PEG [58].

Comparing the spectra of CS/PEG film and Lap with that of CS/PEG/Lap film, some significant differences can be identified. CS/PEG/Lap spectrum shows the peaks corresponding to both CS/PEG film and Lap, but with a higher intensity due to the various interactions which are formed between the three components of the sample. The broadband attributed to OH and NH vibrations at 3400 cm^−1^ in CS/PEG film is slightly shifted to a lower value of wavenumber, namely to around 3368 cm^−1^. This shifting is due to hydrogen bonds and electrostatic interactions between the amino and hydroxyl groups of CS with Si–OH and Mg–OH from the Lap structure [59,60]. Moreover, a significant increase in peak intensity is observed for N–H stretching vibration from –NH_2_ groups at 1568 cm^−1^ as a result of the additional hydrogen bonds between clay particles and CS chains [61]. The stretching vibration of Si–O and Mg–O bands at 966 cm^−1^ and 646 cm^−1^ in the Lap spectrum are shifted to 991 cm^−1^ and 650 cm^−1^, respectively, in CS/PEG/Lap film spectrum. Moreover, the stretching vibration of the C–O–C group at 1026 cm^−1^ in the CS spectrum is shifted to 1068 cm^−1^ in CS/PEG/Lap film spectrum. These results indicate the formation of hydrogen bonding between the three components.

**Figure 4:**
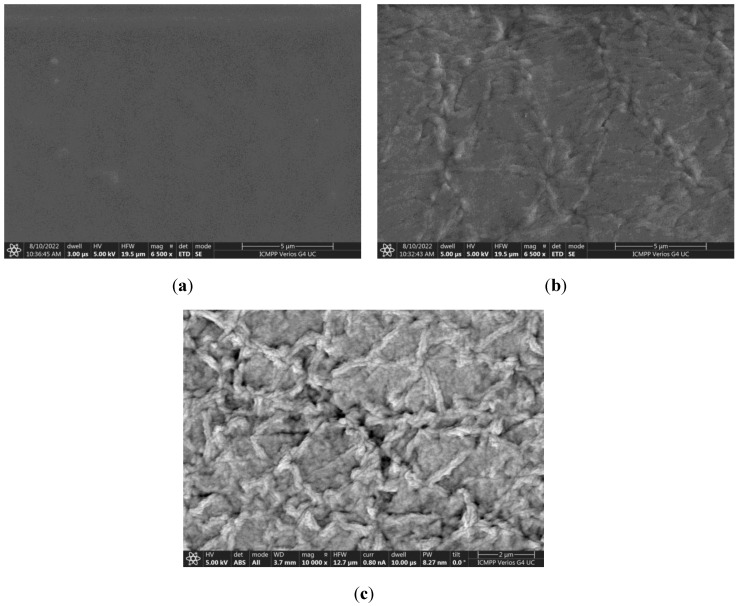
SEM microphotographs of C1 (free of Lap) (**a**) and C5 (with Lap) films were obtained at magnifications of 6500× (**b**) and 10,000× (**c**).

The morphological and texture analysis of the films surface was performed by SEM and POM. Figure 4 shows the SEM images for CS/PEG films with and without Lap.

The PEG films containing chitosan showed a flat surface, with some imperfections from place to place, reminiscent of possible aggregates (Figure 4a). This topography is in line with a fine dispersion of the chitosan chains into the PEG matrix, attributable to the good structural compatibility of these two polymers, i.e., a linear structure and the presence of oxygen heteroatoms with the ability to form H-bonds [62]. This observation is in concordance with those reported in the literature [63,64]. The incorporation of clay greatly modified the morphology of the films, which becomes very rough (Figure 4b). It can be assumed that this is a consequence of the clay encapsulation into the polymer matrix, in which the “house of cards” configuration of clay platelets is surrounded by PEG and chitosan chains, giving continuous films (Figure 4c). A deeper view of the films by polarized light microscopy revealed that PEG-chitosan films are slightly birefringent, with a banded texture, pointing for a crystallinity degree.

**Figure 5:**
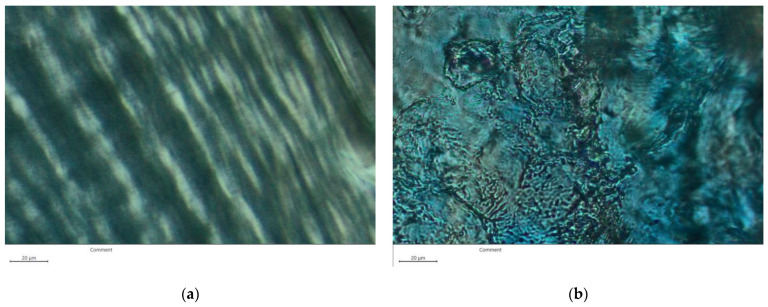
POM images of (**a**) C1 and (**b**) C5 films.

Nevertheless, the spherulites characteristic of PEG crystallization were not present, possibly due to the fine dispersion of chitosan that suppressed its crystallization pattern [65] (Figure 5a).

The addition of Lap intensified the birefringence, regardless of the clay content, with the dominance of the banded texture of the polymeric matrix (Figure 5b). No characteristic spherulites of Lap crystals were discriminated, showing that they were finely dispersed into the PEG/chitosan matrix. All these demonstrate that the three components of the ternary CS/PEG/Lap blend influence each other by intermolecular forces, resulting in a distinct morphology.

**Figure 6:**
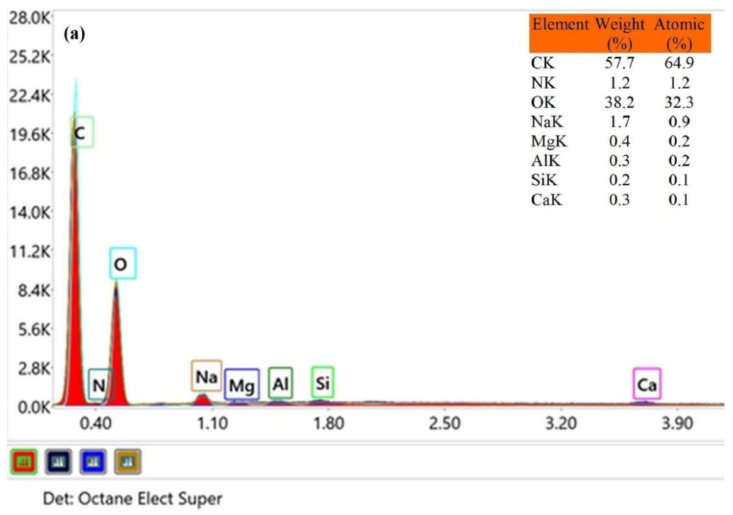

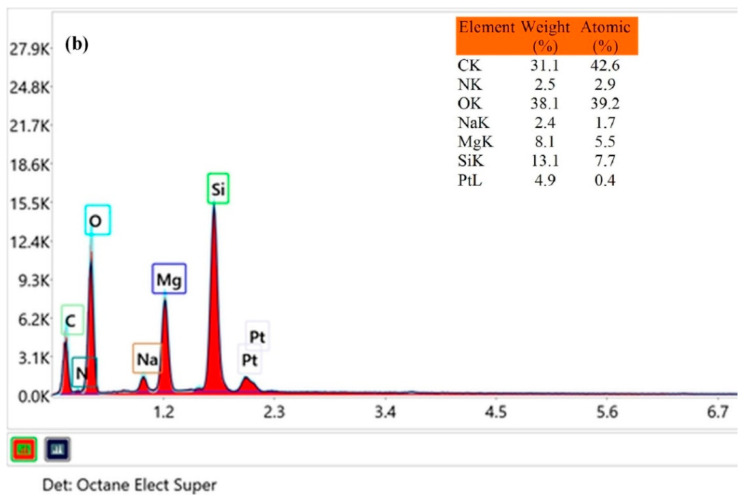
EDX analysis of samples (**a**) C1 and (**b**) C5.

In order to confirm the presence of Lap in CS/PEG/Lap film structure, EDX measurements were performed on different areas of C1 and C5 films. The composition of CS/PEG film (C1) reveals that the main elements are C, O, and N in a percentage of 57.7%, 38.2%, and 1.2% (in weight %), respectively (Figure 6a). The element Na results from the process of adjusting the pH to 7.3 by adding a certain amount of 0.1 M NaOH solution and, the other elements in a percentage less than 1% are due to the presence of some residual elements in the film components. The composition profile of CS/PEG/Lap film (C5) is shown in Figure 6b. The presence of Mg and Al elements in a percentage of 8.1% and 13.1%, respectively, confirms the clay inclusion in the CS/PEG film composition.

### 3.4. Thermal Analysis

**Figure 7:**
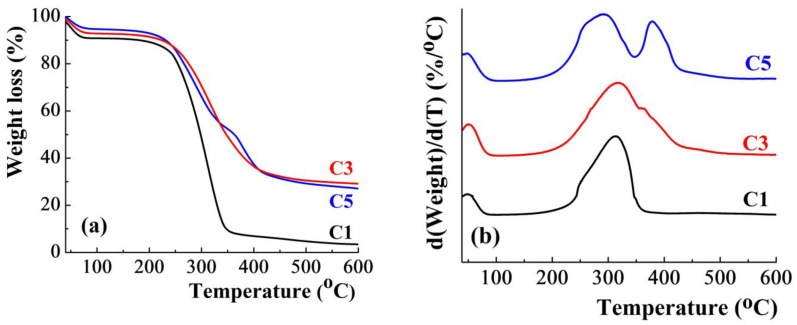
TGA (**a**) and DTG (**b**) curves of CS/PEG films with various amounts of Lap.

Figure 7 shows the thermal gravimetric curves (TGA and DTG) for films C1, C3, and C5. For CS and Lap, the curves have not been shown because they are well-known in the literature. According to Neto et al. [66], weight loss for CS films takes place in two stages. In the first stage, between 60 °C and about 80 °C, CS film loses 10% of its mass. The second stage begins at 240 °C and extends up to 380 °C with a weight loss of 41.4% as a result of dehydration of the saccharide rings and decomposition of the acetylated/deacetylated units of CS. Lap showed a first mass loss of 17.5 % at a temperature lower than 150 °C and, between 150 °C and 800 °C a weight loss of only 6.7% attributed to dehydroxylation [67].

The thermal analysis of C1, C3, and C5 films evidenced, in the first stage below 80 °C, the weight losses of 9%, 7%, and 5%, respectively, due to the loss of adsorbed water (Figure 7a,b). For C1 film, the temperature range for the second stage of thermal degradation was ~225–360 °C and the weight loss was about 79%. The mass loss of C1 was almost double compared to the data reported by Neto et al. [66] for CS films, proving that the incorporation of PEG molecules with low molecular weight into a physical CS network worsens the thermal stability of the CS film.

In the temperature range of ~225–410 °C, the C3 film (31.9% Lap) showed the second stage in the thermal degradation with a weight loss of about 51%. Further increase of Lap amount to 43.8% (C5), changes the appearance of the TGA curve. Thereby, the weight loss for sample C5 takes place in three stages. First, below 80 °C, the second stage between 200 °C and 350 °C (with weight loss of 38%), and the third step in the temperature range of 325–410 °C (with a weight loss of 22%). The existence of the two stages of degradation between 200 °C and 410 °C is probably due to the two phases that form as a result of a higher Lap content. In the films with c_Lap_ > 38.5%, the clay particles form a “house of cards” network inside of which the chitosan chains are intercalated, leading to separation into a clay-rich phase and chitosan-rich one (this separation was also observed from SEM microphotographs). Thereby, the stage of decomposition from 200 °C to 350 °C could be attributed to a mass loss due to the decomposition of the CS network, and, the stage between 325 °C and 410 °C could be related to the decomposition of the Lap network. In the DTG curve of C3 film, a shoulder can be identified at ~375 °C. The films C1, C3, and C5 lose 50% of their weight at 298 °C, 347 °C, and 363 °C, respectively, proving that the incorporation of Lap in CS/PEG films leads to a delay in mass loss.

**Figure 8:**
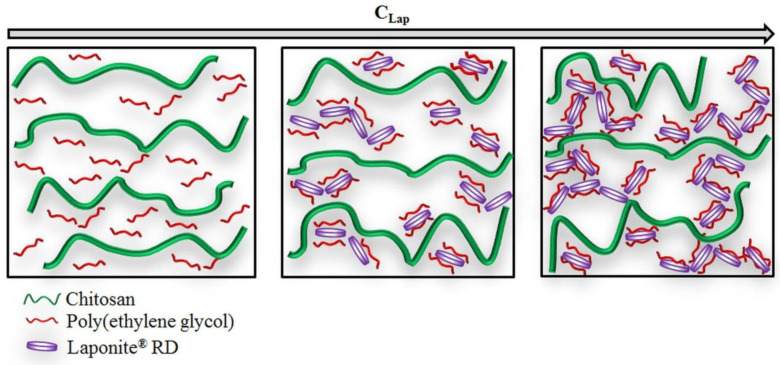
Schematic representation of film structures with various clay concentrations.

Based on the SEM, POM, and TGA observations, the microstructure of the films without clay, with a medium and high concentration of Lap is shown in Figure 8.

In the absence of Lap, the PEG molecules are distributed among the CS chains and hydrogen bonds are formed between the two polymers [68]. The addition of Lap determines the formation of the hydrogen bonds between PEG and CS chains, on one hand, and the formation of PEG-Lap and CS-Lap interactions, on the other hand. A “house of cards” configuration of clay platelets on which are adsorbed PEG chains are formed at higher concentration of Lap, and CS chains remain embedded in this network. The adsorption of PEO chains onto Lap platelets from aqueous dispersion can be explained by the following interactions: (i) ion−dipole interactions between Na^+^ ions from the interlayer region and the nonionic polymer sequences; (ii) van der Waals interactions; (iii) entropy effect; (iv) hydrogen bonds between the surface silanol groups and the ether oxygen from the polymer chains; (v) the hydrophobic interactions between −CH_2_−CH_2_− groups from polymer and the siloxane surface of clay [69,70,71].

### 3.5. Swelling Measurements

**Figure 9:**
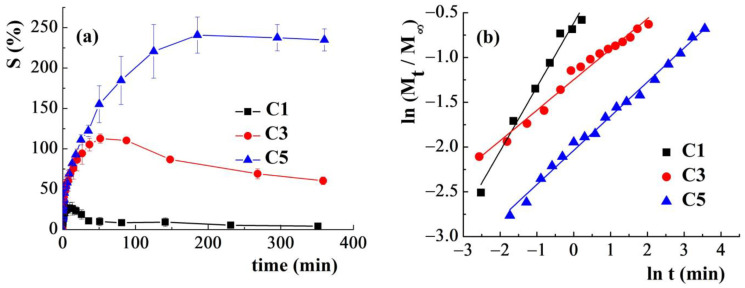
(**a**) Swelling degree, S, vs. immersion time in the water at room temperature and (**b**) the plots of ln(M_t_/M_∞_) as a function of ln t for CS/PEG films with/without Lap.

The addition of Lap significantly modifies the swelling degree of CS/PEG film. The films exhibit a poor water retention capacity (values are lower than 250%) and, after a time which depends on composition, the films lose components and, finally they disintegrate. Figure 9a, it is exemplified the variation of S as a function of time for the films C1, C2, and C3.

It can be observed that the addition of Lap improves the strength of the network formed in the hydrogel. Thereby, the samples C1, C3, and C5 start to disintegrate after about 10 min, 80 min, and 180 min, respectively. The film free of clay shows the lowest degree of swelling and the weakest structure as a result of the not-very-strong physical interactions between CS and PEG. The film with the highest amount of Lap, C5, exhibits the strongest network due to the “house of cards” structure formed by the clay disks covered with adsorbed polymer and the microfibrillar arrangement of chitosan chains. This strong network formed by the clay particles gives the highest stability in water to sample C5, with the film disintegrating more slowly after a long time (180 min). S value increases from 26.8% for C1 to 240.7% for C5.

The diffusion mechanism of water during the swelling was discussed considering the Korsmeyer–Peppas model [72]:

(4) $$\frac{M_{t}}{M_{\infty }}=k\cdot t^{n}\;\frac{M_{t}}{M_{\infty }}<0.6$$

where M_t_ and M_∞_ represent the amount of water adsorbed at time t and at equilibrium, respectively.

k (min^−n^) is the kinetic constant and n represents the diffusional exponent which can give an indication about the diffusion mechanism. For films, as a function of n value, the following ranges can be identified: (i) n = 0.5 when the water diffusion occurs according to the Fickian mechanism; (ii) 0.5 < n < 1 when water molecules diffuse into film by non–Fickian (anomalous) mechanism; (iii) n = 1 for relaxation–controlled transport [73].

The n values lower than 0.5 show a pseudo–Fickian diffusion (or two-stage sorption) in which the water molecules diffuse rapidly in the first minutes in the film, after which the equilibrium value is reached slowly. k and n values for samples C1, C3 and C5 have been determined from the representation of ln(M_t_/M_∞_) as a function of ln t (for M_t_/M_∞_ < 0.6) (Figure 9b).

The water molecules diffuse into film C1 by a non–Fickian mechanism where the diffusion and relaxation rates are comparable and, after about 10 min, the film starts to disintegrate. The films C3 and C5 showed values of n lower than 0.5, specific for pseudo–Fickian diffusion mechanism. This mechanism is still debated in the literature due to the multiple phenomena that can affect diffusion: the relaxation and mobility of polymer chains and water molecules, the polymer–water interactions, the stress caused by the water sorption, etc. Pseudo–Fickian diffusion mechanism could be attributed to the different sizes of the pores and the structural heterogeneity [74] or to the polymer chain rearrangement and the formation of water molecules clusters [75]. The kinetic constant, k, decreases as the amount of Lap increases. The clay addition determines the increase of the strength of the formed networks and of the physical crosslinking density of the film, decreasing the water diffusion speed.

## 4. Conclusions

In the present investigation, some hybrid materials based on CS, PEG, and Lap were prepared by the solution casting method. The rheological properties of starting dispersions were evaluated and discussed. The addition of 2.5% clay to the CS/PEG aqueous dispersions improved the viscoelastic moduli by approximately two orders of magnitude and shifted the limit of the linear viscoelastic range from 0.2 Pa to 10 Pa. The strong interactions developed between components by the addition of clay and the formation of complexes between CS or PEG and Lap led to turbidity increasing and to obtaining more opaque films. The structural characterization by FTIR and EDX has proved the incorporation of clay into CS/PEG film.

A “house of cards” structure of clay platelets, which contains intercalated CS and PEG chains, was revealed for the film with 43.8% Lap. The presence of clay in CS/PEG film delays the loss of mass at high temperatures and increases the film stability in water. Thereby, the film free of Lap and the one with 43.8% Lap loses 50% from their weight at 298 °C and 363 °C, respectively. The films without Lap and those containing 31.9% Lap and 43.8% Lap were stable in water for up to about 10 min, 80 min, and 180 min, respectively. The swelling experiments showed that the water diffusion mechanism changes with addition of Lap from non-Fickian mechanism to pseudo–Fickian one, probably due to the increase in structural heterogeneity of the films with clay. For films with a higher amount of clay, a mechanism involving the formation of a network of clay platelets on which PEG chains are adsorbed, and which includes CS chains has been proposed. The data obtained in the present study exhibit that the films, which combine the properties of CS and PEG with those of Lap, could be potential candidates for obtaining support materials for drug release.

## Figures and Tables

**Table 1:** The composition of the investigated samples.

Sample	Dispersion			Film		
	CS (%)	PEG (%)	Lap (%)	PEG/CS (g/g)	PEG/Lap (g/g)	Lap (%)
C1	0.2	3	0	14.9	-	0
C2	0.2	3	1	14.9	3	23.7
C3	0.2	3	1.5	14.9	2	31.9
C4	0.2	3	2	14.9	1.5	38.5
C5	0.2	3	2.5	14.9	1.2	43.8

**Table 2:** Rheological and swelling parameters of CS/PEG/Lap dispersions and films, respectively.

Sample	G′ ^a^	G″ ^a^	tan δ	η_0_^b^	τ_0_^c^	S ^d^	n ^e^	K ^e^
	(Pa)	(Pa)	(=G″/G′)	(Pa·s)	(Pa)	(%)		(min^−n^)
C1	6.6	1.1	0.17	131.7 ± 3.9	0.21	26.8 ± 6.6	0.72 ± 0.04	0.54 ± 0.03
C2	82.9	17.1	0.21	10,609 ± 99	9.90	–	–	–
C3	322.3	42.4	0.13	13,583 ± 215	13.85	112.8 ± 5.7	0.34 ± 0.01	0.29 ± 0.005
C4	564.1	65.1	0.12	23,410 ± 344	27.47	–	–	–
C5	657.7	80.1	0.12	28,875 ± 711	16.57	240.7 ± 22.3	0.38 ± 0.01	0.13 ± 0.002

^a^ values provided from the frequency sweep measurements at 5 rad/s; ^b^ estimated with the three-parameter Carreau model [46]: $\eta \left(\dot{\gamma }\right)=\eta_{0}/{\left(1+{\left(\lambda \cdot \dot{\gamma }\right)}^{2}\right)}^{\frac{1-n}{2}}$ where η_0_ is zero shear viscosity, λ and n represent the relaxation time and power index, respectively, which are characteristic of the material; ^c^ determined as the τ value at which the η_app_ suddenly decreases (Figure 1f); ^d^ calculated with Equation (2); ^e^ n and k were determined with Korsmeyer-Peppas equation (Equation (4)); M_∞_ was considered the maximum amount of water adsorbed by the film before disintegration.

## Data Availability

The data presented in this study are available on request from the corresponding author.
